# An evaluation of whether a gestational weight gain of 5 to 9 kg for obese women optimizes maternal and neonatal health risks

**DOI:** 10.1186/s12884-019-2273-z

**Published:** 2019-04-11

**Authors:** Abaigeal M. Thompson, James A. Thompson

**Affiliations:** 1 School of Medicine, University of Texas, Rio Grande Valley, 1201 West University Drive, Edinburg, TX 78539 USA; 2 College of Veterinary Medicine and Biomedical Science, Texas A&M University, College Station, TX 77843-4475 USA

**Keywords:** Gestational weight gain, Body mass index, Neonatal, Maternal, Adverse outcomes

## Abstract

**Background:**

Maternal obesity has a wide range of health effects on both the pregnant woman and developing fetus. The clinical significance of these disorders, combined with a dramatically increasing prevalence of obesity among pregnant women has precipitated a major health crisis in the United States. The most commonly used recommendations for gestational weight gain were established by the Institute of Medicine (IOM) in 2009 and have become well known and often adopted. The authors of the IOM report acknowledged that the recommended gestational weight gain of 5 to 9 kg for obese women whose body mass index was greater than 30 kg/m^2^ was based on very little empirical evidence. The objective of this study was to evaluate whether a 5 to 9 kg weight gain, for obese women, optimized a set of maternal and neonatal health outcomes.

**Methods:**

Data containing approximately 12,000,000 birth records were obtained from the United States Natality database for the years 2014 to 2016. A Bayesian modeling approach was used to estimate the controlled direct effects of pre-pregnancy body mass index and gestational weight gain.

**Results:**

Obese women gaining less than 5 kg during pregnancy had reduced maternal risks for gestational hypertension, eclampsia, induction of labor and Caesarian section. In contrast, maternal gestational weight gain of less than 5 kg was associated with increased risks for multiple adverse neonatal outcomes with macrosomia the exception. Obese women who gained more than 9 kg during pregnancy had increased risk for multiple maternal and neonatal adverse outcomes.

**Conclusions:**

Obese women who were observed to gain less than 5 kg during gestation had reduced odds of several peripartum disorders. However, this lower gestational weight gain was associated with an increase in multiple risks for the neonate.

**Electronic supplementary material:**

The online version of this article (10.1186/s12884-019-2273-z) contains supplementary material, which is available to authorized users.

## Background

Maternal obesity has a wide range of health effects on both the pregnant woman and developing fetus [1, 2]. The clinical significance of these disorders, combined with a dramatically increasing prevalence of obesity among pregnant women has precipitated a major health crisis in the United States [3]. Obesity during pregnancy is often attributed to the interplay of pre-pregnancy obesity and gestational weight gain (GWG) and while obesity established prior to pregnancy is a critical concern, it is not preventable when a pregnant woman first seeks prenatal care. As a result of this limitation, addressing GWG becomes the primary preventative option during prenatal care [4].

Maternal obesity causes pregnancy-onset metabolic conditions including gestational diabetes mellitus (GDM), gestational hypertension and pre-eclampsia/eclampsia [5]. These prepartum conditions increase the risks for severe maternal morbidities (SMM) the unintended outcomes of the process of labor and delivery that result in significant short-term or long-term consequences to a woman’s health [6]. Severe maternal morbidities include maternal blood transfusions, admission to an Intensive Care Unit (ICU), ruptured uterus, unplanned hysterectomy and perineal lacerations. The list of SMM also often includes caesarian section as a preferably prevented consequence of obesity. The neonatal risks associated with maternal obesity, pre-partum metabolic conditions and SMM include mortality and with multiple long term consequences [1, 7, 8].

In the United States, the most commonly used recommendations for GWG were established by the Institute of Medicine (IOM) in 2009 [4]. Guidelines were developed for six categories of pre-pregnancy body mass index (ppBMI) commonly used by the World Health Organization (WHO). While publishing these guidelines, the authors acknowledged a number of limitations including a lack of data to identify optimal GWG at higher levels of obesity. As a result, the guidelines provided the same recommendation for GWG of 5 to 9 kg for women classified by the WHO BMI classifications as Obese I (BMI of 30.00–34.99 kg/m^2^), Obese II (BMI of 35.00–39.99 kg/m^2^) or Obese III (BMI of ≥40.00 kg/m^2^) [4]. To resolve the deficiency of empirical evidence supporting these guidelines, the IOM recommended collecting more data and making these data available to researchers [4]. This recommendation has been followed. In the United States, state laws require birth certificates to be completed for all births, and Federal law mandates national collection and publication of births and other vital statistics data. The National Vital Statistics System, the Federal compilation of this data, is the result of the cooperation between the National Center for Health Statistics and the states to provide access to statistical information from birth certificates. The data collection has included maternal metabolic conditions, SMM and adverse neonatal outcomes. The relationships between these and GWG can now be evaluated and, potentially, updated on data-based criteria. Obesity related conditions that are recorded in the birth database include pregnancy onset metabolic conditions, SMM and a variety of fetal viability assessments. The objective of this study was to utilize the national vital statistics database to evaluate whether a 5 to 9 kg weight gain, for obese women, optimized a set of maternal and neonatal health outcomes.

## Methods

Data containing approximately 12,000,000 birth records were obtained from the United States Natality database for the years 2014 to 2016. The entire database was downloaded and the following variables were extracted for analysis: maternal race/ethnicity, plurality, BMI class and gestational weight gain. The BMI classification used the six category WHO classification. The following maternal conditions and events were extracted: GDM, gestational hypertension, pre-eclampsia/eclampsia, occurrences of labor induction and delivery by caesarian section, blood transfusion, perineal laceration, uterine rupture, unplanned hysterectomy and ICU admission. The following fetal conditions and events were extracted: birth weight, obstetrical estimate of gestation length, five-minute Apgar score, admission to NICU, whether the infant was ventilated for at least six minutes, and whether the baby was alive at the time of the birth being recorded.

The maternal weight gain was standardized to 40 weeks of gestation using internal standardization. Gestational weight gain was then classified as being in the recommended range or above or below the range according to the cut-points as recommended by IOM. For the purpose of classification, the following definitions were used: Extremely low birth wieht (ELBW) was birth weight less than 1000 g, very low birth weight (VLBW) was birth at less than 1500 g, low birth weight (LBW) was birth at less than 2500 g, very preterm birth (VPTB) was birth before 32 weeks of gestation and preterm birth (PTB) was birth before 37 weeks of gestation.

A Bayesian modeling approach was used to perform a 3-way decomposition of total effects into direct, indirect and interactive effects [9, 10]. For each of i disorders, case counts were cross-tabulated by j = 6 ppBMI classes (underweight, normal weight, overweight and obese I,II and III) and k = 3 GWG classes (less than recommended, recommended and greater than recommended). For each row in the table Y_ijk_ was the count of cases, at birth, and n_ijk_, the count of births. The counts, Y_ijk_ were modeled as independent Binomial distributions conditional on an unknown rate parameter (μ_ijk_).

$$ {\mathrm{Y}}_{\mathrm{ijk}}\sim \mathrm{Binomial}\ \left({\upmu}_{\mathrm{ijk}},{\mathrm{n}}_{\mathrm{ijk}}\right) $$

The logit of the rate parameter was then provided a vague normal prior (R_ijk_) with a mean = 0 and variance = 100, for each combination of GWG and ppBMI.

$$ \mathrm{Logit}\ \left({\upmu}_{\mathrm{ijk}}\right)={\mathrm{R}}_{\mathrm{ijk}} $$

To aid in identifiability, the R_ijk_ were then standardized to the stratum for which the estimate for the normal weight ppBMI with the observed recommended weight gain was set to zero. The implementation used Markov Chain Monte Carlo (MCMC) and the software OpenBUGS 3.2.3 [11]. A burn-in of 5000 iterations was allowed then the next 10,000 iterations were sampled for the posterior distribution. Convergence was evaluated by observing convergence of separate chains with diverse starting values. The median, the lower 2.5% limit, the median and the upper 97.5% limit were all drawn from the complete posterior distributions. The authors refer to the interval from the 2.5 percentile to 97.5 percentile values as the 95% Bayesian credible interval. When the lower bound of this credible interval is greater than 1, the value for the Bayesian exceedance probability would be greater than 95% which would be relatively analogous to a frequentist *p*-value of less than 5% for a 2-tailed test. The posterior predictive *p* value was used to evaluate all odds ratios with a p-value < 0.05 referred to as statistically significant [12]. Results were presented and discussed for obese women. The model and data are provided for readers in additional materials (Additional file 1).

## Results

The database recorded 11,529,069 singleton births. Incomplete records that were excluded numbered 568,351 that were missing ppBMI, 139,657 that were missing maternal weight gain, 4385 that were missing gestation length estimates and 5171 that were missing birth weights. The study modeled data from 10,811,496 birth records that were cross-stratified into six ppBMI categories and three GWG categories with the number and percent of each combination among obese women reported in Table 1. These records included 2,716,860 women whose ppBMI was classified as obese I, II or III.

**Table 1:** Cross stratification frequency table with counts of births and percent of births

	Pre-pregnancy Body Mass Index					
	Underweight BMI < 18.5	Normal Weight BMI 18.5–24.9	Overweight BMI 25.0–29.9	Obese I BMI 30.0–34.9	Obese II BMI 35.0–39.9	Obese III BMI > 40.0
Gestational weight gain						
< Recommended	115,003 1.1%	1,216,654 11.3%	385,373 3.6%	238,594 2.2%	174,923 1.6%	163,436 1.5%
Recommended	158,559 1.5%	1,572,849 14.6%	621,887 5.8%	252,205 2.3%	136,807 1.3%	94,799 0.9%
> Recommended	123,477 1.1%	2,111,670 19.5%	1,789,164 16.6%	1,013,805 9.4%	408,098 3.8%	234,193 2.2%

The overall modeling was performed with the baseline arbitrarily set at the combination of normal weight ppBMI and the currently recommended GWG, for each disorder. These model results are presented in Tables 2, 3 and 4. Table 2 presents the baseline incident risk as a percent probability and Tables 3 and 4 as the odds ratios for combinations of ppBMI and GWG with adverse maternal outcomes and adverse neonatal outcomes, respectively.

**Table 2:** Baseline incidence risk (%) at normal pre-pregnancy body mass index and recommended gestational weight gain

Disorder	Baseline incidence risk (%)
Maternal outcomes	
Gestational diabetes	3.24 (3.21, 3.27)
Gestational hypertension	2.50 (2.48, 2.52)
Eclampsia	0.11 (0.11, 0.12)
Labor induction	14.6 (14.6, 14.7)
Caesarian section	15.3 (15.3, 15.4)
Maternal transfusion	0.24 (0.23, 0.24)
Perineal laceration	1.05 (1.03, 1.07)
Ruptured uterus	0.02 (0.02, 0.03)
Unplanned hysterectomy	0.03 (0.02, 0.03)
Admission to ICU	0.1 (0.1, 0.11)
Neonatal outcomes	
Extremely low birth weight	0.22 (0.22, 0.23)
Very low birth weight	0.55 (0.54, 0.57)
Low birth weight	5.28 (5.25, 5.32)
Macrosomia	5.3 (5.26, 5.33)
Very pre-term birth	0.64 (0.63, 0.66)
Pre-term birth	5.76 (5.72, 5.8)
Low APGAR score	0.35 (0.34, 0.36)
Admission to neonatal ICU	5.71 (5.67, 5.74)
Ventilated for > 6 min	0.73 (0.71, 0.74)
Neonatal mortality	0.22 (0.21, 0.23)

**Table 3:** Model results (odds ratios) for maternal outcomes by category of pre-pregnancy body mass index and gestational weight gain

Pre-pregnancy body mass index		Obese I			Obese II			Obese III	
Gestational weight gain	< 5 kg	5–9 kg	> 9 kg	< 5 kg	5–9 kg	> 9 kg	< 5 kg	5–9 kg	> 9 kg
Gestational diabetes	3.5 (3.4, 3.5)^a^	3.3 (3.3, 3.4) ^a^	2.5 (2.5, 2.5) ^a^	4.3 (4.2, 4.3) ^a^	4.0 (3.9, 4.0) ^a^	3.3 (3.2, 3.3) ^a^	5.3 (5.2, 5.4) ^a^	4.7 (4.6, 4.8) ^a^	4.3 (4.2, 4.4) ^a^
Gestational hypertension	2.1 (2, 2.1) ^a^	2.5 (2.5, 2.6) ^a^	3.8 (3.8, 3.9) ^a^	3.0 (2.9, 3.0) ^a^	3.6 (3.6, 3.7) ^a^	5.3 (5.2, 5.3) ^a^	4.5 (4.4, 4.5) ^a^	5.4 (5.3, 5.5) ^a^	7.3 (7.2, 7.4) ^a^
Eclampsia	2.0 (1.8, 2.2) ^a^	2.1 (1.9, 2.3) ^a^	3.4 (3.2, 3.6) ^a^	2.5 (2.3, 2.8) ^a^	2.9 (2.6, 3.2) ^a^	4.6 (4.3, 4.9) ^a^	3.5 (3.2, 3.9) ^a^	4.6 (4.1, 5.0) ^a^	6.1 (5.7, 6.5) ^a^
Labor induction	1.2 (1.2, 1.2) ^a^	1.3 (1.3, 1.3) ^a^	1.4 (1.4, 1.4) ^a^	1.4 (1.4, 1.4) ^a^	1.5 (1.5, 1.5) ^a^	1.5 (1.5, 1.5) ^a^	1.6 (1.6, 1.6) ^a^	1.6 (1.5, 1.6) ^a^	1.6 (1.6, 1.6) ^a^
Caesarian section	1.5 (1.5, 1.5) ^a^	1.7 (1.7, 1.7) ^a^	2.0 (2.0, 2.0) ^a^	1.9 (1.8, 1.9) ^a^	2.1 (2.1, 2.1) ^a^	2.6 (2.5, 2.6) ^a^	2.6 (2.6, 2.7) ^a^	2.9 (2.9, 3.0) ^a^	3.4 (3.4, 3.4) ^a^
Maternal transfusion	1.3 (1.2, 1.4) ^a^	1.2 (1.1, 1.3) ^a^	1.2 (1.1, 1.2) ^a^	1.2 (1.1, 1.4) ^a^	1.1 (1.0, 1.3) ^a^	1.2 (1.1, 1.3) ^a^	1.3 (1.2, 1.4) ^a^	1.2 (1.1, 1.4) ^a^	1.3 (1.2, 1.5) ^a^
Perineal laceration	0.5 (0.4, 0.5) ^b^	0.5 (0.5, 0.5) ^b^	0.6 (0.5, 0.6) ^b^	0.4 (0.4, 0.4) ^b^	0.4 (0.4, 0.4) ^b^	0.4 (0.4, 0.5) ^b^	0.3 (0.3, 0.3) ^b^	0.3 (0.3, 0.4) ^b^	0.3 (0.3, 0.3) ^b^
Ruptured uterus	1.4 (1.1, 1.8) ^a^	1.1 (0.9, 1.5)	1.3 (1.1, 1.5) ^a^	1.4 (1.1, 1.8) ^a^	1.1 (0.8, 1.5)	1.2 (1.0, 1.5) ^a^	1.6 (1.2, 2.1) ^a^	1.3 (0.9, 1.9)	1.4 (1.1, 1.8) ^a^
Unplanned hysterectomy	1.8 (1.4, 2.1) ^a^	1.7 (1.4, 2.1) ^a^	1.5 (1.3, 1.8) ^a^	1.5 (1.1, 1.9) ^a^	1.3 (1.0, 1.7) ^a^	1.6 (1.3, 1.9) ^a^	1.9 (1.5, 2.3) ^a^	1.7 (1.3, 2.3) ^a^	1.9 (1.5, 2.3) ^a^
Admission to ICU	1.4 (1.2, 1.5)	1.3 (1.1, 1.4) ^a^	1.3 (1.3, 1.4) ^a^	1.5 (1.3, 1.7) ^a^	1.3 (1.1, 1.5) ^a^	1.5 (1.4, 1.7) ^a^	1.7 (1.5, 2.0) ^a^	1.6 (1.4, 1.9) ^a^	2.6 (2.4, 2.9) ^a^

^a^ Odds ratio is significantly elevated relative to ideal BMI and recommended weight gain

^b^ Odds ratio is significantly lower relative to ideal BMI and recommended weight gain

**Table 4:** Model results (odds ratios) for neonatal outcomes by category of pre-pregnancy body mass index and gestational weight gain (GWG)

Pre-pregnancy body mass index		Obese I			Obese II			Obese III	
Gestational weight gain	< 5 kg	5–9 kg	> 9 kg	< 5 kg	5–9 kg	> 9 kg	< 5 kg	5–9 kg	> 9 kg
Extremely low birth weight	4.4 (4.2, 4.6) ^a^	2.4 (2.2, 2.5) ^a^	2.5 (2.4, 2.6) ^a^	4.3 (4.1, 4.6) ^a^	2.6 (2.4, 2.8) ^a^	3.2 (3.1, 3.4) ^a^	4.5 (4.2, 4.7) ^a^	2.8 (2.6, 3.1) ^a^	4.0 (3.8, 4.3) ^a^
Very low birth weight	3.2 (3.1, 3.3) ^a^	2.0 (1.9, 2.1) ^a^	2.1 (2, 2.1) ^a^	3.1 (2.9, 3.2) ^a^	2.1 (2.0, 2.2) ^a^	2.6 (2.5, 2.7) ^a^	3.2 (3.1, 3.4) ^a^	2.3 (2.2, 2.5) ^a^	3.2 (3.1, 3.3) ^a^
Low birth weight	1.6 (1.5, 1.6) ^a^	1.2 (1.2, 1.3) ^a^	1.0 (1.0, 1.1) ^a^	1.4 (1.4, 1.4) ^a^	1.1 (1.1, 1.2) ^a^	1.1 (1.1, 1.2) ^a^	1.3 (1.3, 1.4) ^a^	1.2 (1.2, 1.2) ^a^	1.3 (1.3, 1.3) ^a^
Macrosomia	1.1 (1.1, 1.1) ^a^	1.5 (1.5, 1.5) ^a^	2.6 (2.5, 2.6) ^a^	1.5 (1.5, 1.5) ^a^	2.0 (2.0, 2.0) ^a^	3.1 (3.1, 3.1) ^a^	1.9 (1.9, 2.0) ^a^	2.6 (2.5, 2.6) ^a^	3.6 (3.6, 3.6) ^a^
Very pre-term birth	3.0 (2.9, 3.1) ^a^	1.9 (1.8, 2.0) ^a^	2.0 (1.9, 2.0) ^a^	2.9 (2.8, 3.0) ^a^	2.0 (1.9, 2.1) ^a^	2.4 (2.3, 2.5) ^a^	3.0 (2.9, 3.1) ^a^	2.2 (2.0, 2.3) ^a^	3.0 (2.9, 3.1) ^a^
Pre-term birth	1.6 (1.6, 1.7) ^a^	1.5 (1.5, 1.5) ^a^	1.5 (1.4, 1.5) ^a^	1.6 (1.6, 1.6) ^a^	1.6 (1.5, 1.6) ^a^	1.7 (1.7, 1.7) ^a^	1.7 (1.7, 1.8) ^a^	1.7 (1.7, 1.8) ^a^	2.0 (2.0, 2.1) ^a^
Low APGAR score	2.2 (2.1, 2.3) ^a^	1.6 (1.5, 1.7) ^a^	1.7 (1.6, 1.8) ^a^	2.3 (2.2, 2.5) ^a^	1.6 (1.5, 1.8) ^a^	2.1 (2.0, 2.2) ^a^	2.5 (2.4, 2.7) ^a^	2.2 (2.0, 2.4) ^a^	2.8 (2.7, 3.0) ^a^
Admission to neonatal ICU	1.4 (1.4, 1.5) ^a^	1.3 (1.3, 1.4) ^a^	1.5 (1.5, 1.5) ^a^	1.4 (1.4, 1.5) ^a^	1.4 (1.4, 1.5) ^a^	1.7 (1.7, 1.7) ^a^	1.6 (1.6, 1.7) ^a^	1.7 (1.7, 1.8) ^a^	2.1 (2.1, 2.2) ^a^
Ventilated for > 6 min	1.8 (1.7, 1.9) ^a^	1.4 (1.3, 1.5) ^a^	1.7 (1.6, 1.7) ^a^	1.9 (1.8, 2.0) ^a^	1.7 (1.6, 1.8) ^a^	2.0 (1.9, 2.1) ^a^	2.2 (2.1, 2.3) ^a^	2.2 (2.1, 2.3) ^a^	2.7 (2.6, 2.8) ^a^
Neonatal mortality	2.5 (2.3, 2.7) ^a^	1.6 (1.5, 1.7) ^a^	1.6 (1.5, 1.6) ^a^	2.4 (2.2, 2.6) ^a^	1.6 (1.4, 1.7) ^a^	1.8 (1.7, 1.9) ^a^	2.5 (2.3, 2.7) ^a^	1.9 (1.7, 2.1) ^a^	2.2 (2, 2.3) ^a^

^a^Odds ratio is significantly elevated relative to ideal BMI and recommended weight gain

**Table 5:** Odds ratios for women classified as obese I. Odds ratios are for the class of gestational weight gain (less than 5 kg and more than 9 kg) relative to 5 to 9 kg

	Gestational Weight Gain < 5.0 kg	Gestational Weight Gain > 9.0 kg
Maternal Conditions		
Gestational diabetes	1.04 (1.02, 1.06) ^a^	0.76 (0.75, 0.77) ^b^
Gestational hypertension	0.82 (0.80, 0.84)^b^	1.52 (1.49, 1.55) ^a^
Eclampsia	0.93 (0.83, 1.04)	1.59 (1.46, 1.73) ^a^
Induction of labor	0.97 (0.96, 0.99) ^b^	1.09 (1.08, 1.11) ^a^
Caesarian Section	0.88 (0.87, 0.90) ^b^	1.21 (1.20, 1.22) ^a^
Maternal transfusion	1.08 (0.98, 1.20)	1.02 (0.94, 1.11)
Perineal laceration	0.98 (0.90, 1.05)	1.16 (1.09, 1.23) ^a^
Ruptured uterus	1.24 (0.89, 1.71)	1.12 (0.87, 1.47)
Unplanned hysterectomy	1.01 (0.78, 1.30)	0.89 (0.73, 1.10)
Admission to Intensive Care Unit	1.07 (0.92, 1.25)	1.07 (0.94, 1.20)
Neonatal Conditions		
Extremely low birth weight	1.87 (1.74, 2.00) ^a^	1.07 (1.01, 1.14) ^a^
Very low birth weight	1.58 (1.51, 1.66) ^a^	1.02 (0.98, 1.07)
Low birth weight	1.27 (1.24, 1.29) ^a^	0.84 (0.83, 0.86) ^b^
Macrosomia	0.73 (0.71, 0.74) ^b^	1.72 (1.69, 1.75) ^a^
Very pre-term delivery	1.61 (1.54, 1.69) ^a^	1.06 (1.02, 1.10) ^a^
Preterm delivery	1.10 (1.08, 1.12) ^a^	0.96 (0.95, 0.98) ^b^
Low Apgar score	1.40 (1.31, 1.05) ^a^	1.07 (1.01, 1.13) ^a^
Admission to Neonatal Intensive Care Unit	1.07 (1.05, 1.09) ^a^	1.09 (1.08, 1.11) ^a^
Ventilation for more than 6 min	1.27 (1.21, 1.34) ^a^	1.18 (1.13, 1.23) ^a^
Neonatal mortality	1.56 (1.43, 1.70) ^a^	0.97 (0.91, 1.05)

^a^ Odds ratio is significantly elevated relative to gestational weight gain of 5 to 9 kg

^b^ Odds ratio is significantly lower relative to gestational weight gain of 5 to 9 kg

For women who were obese I, GWG less than 5 kg was associated with a significantly decreased risk for gestational hypertension, induction of labor and Caesarian section and a significantly increased risk of gestational diabetes. The neonates born to these women had significantly increased risk for extremely low birth weight, very low birth weight, low birth weight, very preterm delivery, preterm delivery, low Apgar score, admission to Neonatal Intensive Care Unit, ventilation for more than 6 min and neonatal mortality and a significantly reduced risk for macrosomia. For women whose ppBMI was classified as Obese I, gaining more weight than 9 kg was associated with an increased risk for gestational hypertension, eclampsia, induction of labor, Caesarian section and perineal laceration. The neonates born to these women had increased risk for extremely low birth weight, macrosomia, very pre-term delivery, low Apgar score, admission to Neonatal Intensive Care Unit and ventilation for more than 6 min and significantly reduced risk for low birth weight and preterm delivery (Table 5).

**Table 6:** Odds ratios for women classified as obese II. Odds ratios are for the class of gestational weight gain (less than 5 kg and more than 9 kg) relative to 5 to 9 kg

	Gestational Weight Gain < 5.0 kg	Gestational Weight Gain > 9.0 kg
Maternal Conditions		
Gestational diabetes	1.08 (1.06, 1.10) ^a^	0.83 (0.81, 0.84) ^b^
Gestational hypertension	0.81 (0.79, 0.83) ^b^	1.44 (1.41, 1.47) ^a^
Eclampsia	0.88 (0.77, 1.00) ^b^	1.61 (1.45, 1.79) ^a^
Induction of labor	0.99 (0.97, 1.00) ^b^	1.05 (1.03, 1.06) ^a^
Caesarian Section	0.88 (0.87, 0.90) ^b^	1.22 (1.20, 1.23) ^a^
Maternal transfusion	1.09 (0.96, 1.25)	1.05 (0.94, 1.19)
Perineal laceration	1.01 (0.91, 1.13)	1.13 (1.03, 1.25) ^a^
Ruptured uterus	1.27 (0.84, 1.98)	1.11 (0.77, 1.63)
Unplanned hysterectomy	1.12 (0.78, 1.62)	1.22 (0.90, 1.69)
Admission to Intensive Care Unit	1.14 (0.94, 1.37)	1.17 (1.00, 1.38) ^a^
Neonatal Conditions		
Extremely low birth weight	1.68 (1.55, 1.84) ^a^	1.26 (1.16, 1.36) ^a^
Very low birth weight	1.45 (1.36, 1.54) ^a^	1.21 (1.15, 1.28) ^a^
Low birth weight	1.22 (1.19, 1.26) ^a^	0.99 (0.97, 1.02)
Macrosomia	0.75 (0.73, 0.77) ^b^	1.55 (1.52, 1.58) ^a^
Very pre-term delivery	1.46 (1.38, 1.55) ^a^	1.21 (1.15, 1.28) ^a^
Preterm delivery	1.03 (1.01, 1.06) ^a^	1.08 (1.05, 1.10) ^a^
Low Apgar score	1.44 (1.31, 1.57) ^a^	1.28 (1.18, 1.39) ^a^
Admission to Neonatal Intensive Care Unit	1.00 (0.97, 1.03)	1.19 (1.16, 1.21) ^a^
Ventilation for more than 6 min	1.10 (1.04, 1.17) ^a^	1.16 (1.09, 1.22) ^a^
Neonatal mortality	1.51 (1.35, 1.69) ^a^	1.13 (1.02, 1.25) ^a^

^a^ Odds ratio is significantly elevated relative to gestational weight gain of 5 to 9 kg

^b^ Odds ratio is significantly lower relative to gestational weight gain of 5 to 9 kg

Gaining less than 5 kg, for obese II women was associated with a significantly decreased risk for gestational hypertension, eclampsia, induction of labor and Caesarian section and a significantly increased risk of gestational diabetes. The neonates born to these women had increased risk for extremely low birth weight, very low birth weight, low birth weight, very preterm delivery, preterm delivery, low Apgar score, ventilation for more than 6 min and neonatal mortality and a significantly reduced risk for macrosomia. For women whose ppBMI was classified as obese II, gaining more weight than 9 kg was associated with a significantly decreased risk of gestational diabetes and a significantly increased risk for gestational hypertension, eclampsia, induction of labor, Caesarian section, perineal laceration and admission to Intensive Care Unit. The neonates born to these women had increased risk for extremely low birth weight, very low birth weight, macrosomia, very pre-term delivery, preterm delivery, low Apgar score, admission to Neonatal Intensive Care Unit, ventilation for more than 6 min and neonatal mortality (Table 6).

**Table 7:** Odds ratios for women classified as obese III. Odds ratios are for the class of gestational weight gain (less than 5 kg and more than 9 kg) relative to 5 to 9 kg

	Gestational Weight Gain < 5.0 kg	Gestational Weight Gain > 9.0 kg
Maternal Conditions		
Gestational diabetes	1.13 (1.10, 1.15)^a^	0.91 (0.89, 0.93) ^b^
Gestational hypertension	0.82 (0.80, 0.84) ^b^	1.35 (1.32, 1.38) ^a^
Eclampsia	0.77 (0.69, 0.87) ^b^	1.33 (1.20, 1.47) ^a^
Induction of labor	1.02 (1.00, 1.04) ^a^	1.01 (1.00, 1.03) ^a^
Caesarian Section	0.90 (0.89, 0.92) ^b^	1.17 (1.15, 1.19) ^a^
Maternal transfusion	1.10 (0.94, 1.27)	1.12 (0.98, 1.29)
Perineal laceration	0.90 (0.79, 1.03)	0.94 (0.83, 1.07)
Ruptured uterus	1.21 (0.79, 1.92)	1.06 (0.70, 1.63)
Unplanned hysterectomy	1.07 (0.75, 1.55)	1.07 (0.76, 1.52)
Admission to Intensive Care Unit	1.06 (0.87, 1.29)	1.58 (1.33, 1.89) ^a^
Neonatal Conditions		
Extremely low birth weight	1.59 (1.45, 1.74) ^a^	1.43 (1.31, 1.57) ^a^
Very low birth weight	1.38 (1.29, 1.48) ^a^	1.36 (1.28, 1.45) ^a^
Low birth weight	1.13 (1.09, 1.17)^a^	1.09 (1.06, 1.12) ^a^
Macrosomia	0.75 (0.73, 0.76) ^b^	1.39 (1.36, 1.43) ^a^
Very pre-term delivery	1.41 (1.32, 1.50) ^a^	1.38 (1.30, 1.47) ^a^
Preterm delivery	0.99 (0.96, 1.02)	1.17 (1.14, 1.20) ^a^
Low Apgar score	1.15 (1.05, 1.25) ^a^	1.29 (1.18, 1.40) ^a^
Admission to Neonatal Intensive Care Unit	0.94 (0.92, 0.97) ^b^	1.23 (1.20, 1.27) ^a^
Ventilation for more than 6 min	1.03 (0.96, 1.10)	1.24 (1.17, 1.31) ^a^
Neonatal mortality	1.30 (1.15, 1.46) ^a^	1.15 (1.02, 1.28) ^a^

^a^Odds ratio is significantly elevated relative to gestational weight gain of 5 to 9 kg

^b^Odds ratio is significantly lower relative to gestational weight gain of 5 to 9 kg

For women whose ppBMI was classified as obese III, gaining less weight, during gestation, than 5 kg was associated with a significantly decreased risk for gestational hypertension, eclampsia and Caesarian section and a significantly increased risk of gestational diabetes and induction of labor. The children born to these women had increased risk for extremely low birth weight, very low birth weight, low birth weight, very pre-term delivery, low Apgar score and neonatal mortality and significantly reduced risk for macrosomia and admission to Neonatal Intensive Care Unit. For women whose ppBMI was classified as obese III, gaining more than 9 kg was associated with a significantly decreased risk of gestational diabetes and significantly increased risk of gestational hypertension, eclampsia, induction of labor, Caesarian section and admission to Intensive Care Unit. The neonates born to these women had increased risk for extremely low birth weight, very low birth weight, low birth weight, macrosomia, very pre-term delivery, preterm delivery, low Apgar score, admission to Neonatal Intensive Care Unit, ventilation for more than 6 min and neonatal mortality (Table 7).

## Discussion

The interaction model implemented in this study is fundamentally similar to employing stratification for confounding control. In causal modeling the estimates can be referred to as the CDE or conditional direct effects because they are estimated at specific levels of the modifying variable(s) [13, 14]. This approach was modified by VanderWeele [9, 15] such that the CDE(s) can be estimated using an interaction model. An interaction model equivalent to the VanderWeele approach is readily implemented under an MCMC Bayesian modeling approach using commonly available statistical software. [11] One distinction is that the inferences derived from the analyses reported here are individual-based and not population-based and are appropriate for a clinician to report to a patient, that patient’s risk. The model provided in the appendix can be modified to estimate the improvement in individual risk and its confidence bounds for a patient prior to becoming pregnant and allowing for modification of both ppBMI and planning on a specific GWG.

The current study supports previous studies that have shown maternal health benefits for obese pregnant women who gain less weight than 5 kg or even lose weight [16–18]. Specifically, odds for gestational hypertension, eclampsia, induction of labor and Caesarian section were reduced by the lower GWG. This was consistent across ppBMI. In contrast to the analyses of maternal outcomes, the analysis of adverse neonatal outcomes does not support gestational weight gains that are less than 5 kg. Much of the literature addressing fetal or neonatal risks is based on birth weights and gestational length and, collectively, the findings are inconsistent [19–21]. The current study reports substantial risks for both underweight and preterm births and the odds for neonatal mortality are elevated 50%. These risks are substantial and the evidence should discourage any lowering of the 5 kg lower limit of the IOM guidelines. However, the recommendation by ACOG to monitor fetal growth and development when an obese woman is trying to lose weight or gain less than 5 kg [22] is supported by the current findings. A demonstration that monitoring fetal growth can also identify the fetuses at risk of neonatal mortality caused by too low gestation weight gain would further strengthen the ACOG recommendation. More research into more specific nutritional advice for women who want to lose weight and simultaneously support appropriate fetal development should be encouraged.

The current study showed that, it is harmful for both the mother and neonate for obese women to gain more than 9 kg, during gestation. With few exceptions, adverse maternal outcomes occurred with greater odds when the limit was exceeded. The exception that was consistent for all ppBMI classes was GDM that occurred with decreased odds at the higher class of GWG. Analyses of adverse neonatal outcomes provided considerable additional support for obese women to gain less than 9 kg, during gestation. With few exceptions, adverse neonatal outcomes occurred with greater odds when the limit was exceeded. The exception was low birth weight and PTD had a significantly sparing effect among obese III women who gained more than 9 kg.

Current results showed that GWG was lower among women who developed GDM but the causal relationship remains obscure. Both steps in lowering GWG from above recommended to recommended and from recommended to less that recommended GWG were associated with increased rates of GDM. If the relationship was causal, the conclusion would be that higher GWG prevented GDM. However, the authors of the IOM guidelines predicted difficulties identifying the causal ordering between GWG and disorders that develop during pregnancy, like GDM, and also advised that conditions diagnosed early in pregnancy and managed effectively, may have reduced risk. As predicted, studies evaluating the effect of GWG on GDM has been shown risks to be both increased [23] and decreased [24] The Committee also accurately predicted that treatment for the condition would alter the pattern of weight gain after diagnosis [25, 26]. More recent studies have evaluated GWG prior to early screening and shown that early in pregnancy, increased GWG does precede the diagnosis of GWG [27–29].

This study is limited by the observational nature of the data. The interaction model implemented in this study only corrects for confounding by variables included in the model not for confounding by unrecorded or omitted variables. The current results on GWG on adverse outcomes are not confounded by ppBMI but may be confounded by treatment variables. More specific causal inferences should be pursued. For example, more specific causal modeling could address how ppBMI and GWG contribute to GDM and adverse effects by GDM causing complicating causes that are certain to include hypertension, eclampsia and macrosomia. Further research could clarify the mediating effect of GDM on the relationships between the components of maternal obesity and adverse outcomes through additional causal factors like hypertension and eclampsia. To this end, the modeling approach implemented in this study can be expanded to include analyses specific to two groups of women, including those who do and those who do not develop GDM. This could be achieved by modeling all possible 3-way interactions among ppBMI, GWG and GDM. When combined with close monitoring for the chance development of GDM, the clinician can be equipped with the most useful nutritional recommendations related to GWG. We anticipate that the recommendations for GWG will vary with both ppBMI and GDM diagnosis and we encourage further research.

## Conclusions

Obese women who were observed to gain less than 5 kg during gestation had reduced odds of several peripartum disorders. However, this lower gestational weight gain was associated with an increase in multiple risks for the neonate.

## Additional file

Additional file 1: Winbugs code and data. This code and these data can be used to repeat the analyses reported here. (DOCX 14 kb)

## Abbreviations

ACOG: American College of Obstetricians and Gynecologists
ELBW: Extremely low birth weight
GDM: Gestational diabetes mellitus
GWG: Gestational weight gain
ICU: Intensive care unit
IOM: Institute of Medicine
IRB: Institutional review board
LBW: Low birth weight
MCMC: Markov Chain Monte Carlo
ppBMI: Pre-pregnancy Body Mass Index
PTB: Preterm birth
SMM: Severe maternal morbidity
VLBW: Very low birth weight
VPTB: Very preterm birth
WHO: World Health Organization
