# Supplementary material for: An evaluation of whether a gestational weight gain of 5 to 9 kg for obese women optimizes maternal and neonatal health risks
Source: BMC Pregnancy Childbirth. 2019 Apr 11;19:126. doi: 10.1186/s12884-019-2273-z (PMC6460820; doi:10.1186/s12884-019-2273-z)
Supplement: Supplementary file 1 — Winbugs code and data. This code and these data can be used to repeat the analyses reported here. (DOCX 14 kb) [file 12884_2019_2273_MOESM1_ESM.docx]

Additional File 1: Winbugs code and Data

model{ for (h in 1:18) {for (i in 1:10){ Y[h,i] ~ dbin(mu[i,h], n[h])

logit(mu[i,h]) <- R[i,BMI[h],GWG[h]] }}

for (i in 1:10){ for (j in 1:6){ hi.gwg[j,i] <- exp(R[i,j,3] - R[i,j,2])

low.gwg[j,i] <- exp(R[i,j,1] - R[i,j,2])

for (k in 1:3) { R[i,j,k] ~ dnorm(0,0.01)

OR[i,j,k] <- exp(R[i,j,k] - R[i,2,2] ) }}}}

inits

list(R=structure(.Data=c(0,0,0,0,0,0,0,0,0,0,0,0,0,0,0,0,0,0,0,0,0,0,0,0,0,0,0,0,0,0,0,0,0,0,0,0,0,0,0,0,0,0,0,0,0,0,0,0,0,0,0,0,0,0,0,0,0,0,0,0,0,0,0,0,0,0,0,0,0,0,0,0,0,0,0,0,0,0,0,0,0,0,0,0,0,0,0,0,0,0,0,0,0,0,0,0,0,0,0,0,0,0,0,0,0,0,0,0,0,0,0,0,0,0,0,0,0,0,0,0,0,0,0,0,0,0,0,0,0,0,0,0,0,0,0,0,0,0,0,0,0,0,0,0,0,0,0,0,0,0,0,0,0,0,0,0,0,0,0,0,0,0,0,0,0,0,0,0,0,0,0,0,0,0,0,0,0,0,0,0),.Dim = c(10,6,3)))

data for women; n= no. of births in that strata; Y = counts of disorders

Y[ ,1] =diab Y[ ,2]=hypertension Y[ ,3]=eclampsia Y[ ,4]=INDL Y[ ,5]=csection Y[ ,6]=MTR Y[ ,7]=PLAC Y[ ,8]=RUPT Y[ ,9]=UHYST Y[ ,10]=AICU;

GWG =1 for < recommended; GWG = 2 for recommended; GWG = 3 for > recommended

ppBMI = 1 underweight; ppBMI = 2 normal weight; ppBMI = 3 overweight; ppBMI = 4 obese I; ppBMI = 5 obese II; ppBMI = 6 for obese III

BMI[] GWG[] n[] Y[ ,1] Y[ ,2] Y[ ,3] Y[ ,4] Y[ ,5] Y[ ,6] Y[ ,7] Y[ ,8] Y[ ,9] Y[ ,10]

1 1 115003 3834 2030 130 14428 14532 338 1106 36 30 141

1 2 158559 3927 3215 165 21738 20381 494 1777 38 39 171

1 3 123477 2731 4572 247 19278 18991 420 1318 32 47 189

2 1 1216654 55960 26537 1451 165462 178787 3386 10283 354 407 1469

2 2 1572849 50998 39306 1760 229861 240974 3722 16516 361 428 1608

2 3 2111670 56691 97787 4464 350286 383609 5812 22643 489 706 2514

3 1 385373 30246 13087 661 58149 69854 1183 2362 130 177 527

3 2 621887 43365 23937 1074 98789 122276 1554 4049 178 255 635

3 3 1789164 88925 116000 4935 321967 399768 4661 14562 435 592 2036

4 1 238594 24730 11931 527 41818 50321 706 1185 77 114 331

4 2 252205 25292 15277 602 45278 58561 688 1285 66 119 326

4 3 1013805 78811 90526 3833 195734 271179 2831 5963 296 424 1394

5 1 174923 21926 12344 492 34393 44096 513 732 57 70 266

5 2 136807 16058 11702 440 27190 37752 368 568 35 49 183

5 3 408098 40403 48525 2102 84151 129196 1156 1918 115 177 638

6 1 163436 24725 16766 644 34856 52892 508 520 60 83 292

6 2 94799 12945 11553 482 19969 32796 269 335 29 45 160

6 3 234193 29443 36962 1583 49868 89351 744 777 75 118 622

END

data for children; n= no. of births in that strata; Y = counts of disorders

Y[ ,1] =apgar Y[ ,2]=ELBW Y[ ,3]=VLBW Y[ ,4] =LBW Y[ ,5]=macro Y[ ,6] = VPTD Y[ ,7]=PTD Y[ ,8] =NICU Y[ ,9] =vent6 Y[ ,10]=mortality GWG =1 for < recommended; GWG = 2 for recommended; GWG = 3 for > recommended

ppBMI = 1 underweight; ppBMI = 2 normal weight; ppBMI = 3 overweight; ppBMI = 4 obese I; ppBMI = 5 obese II; ppBMI = 6 for obese III

BMI[] GWG[] n[] Y[ ,1] Y[ ,2] Y[ ,3] Y[ ,4] Y[ ,5] Y[ ,6] Y[ ,7] Y[ ,8] Y[ ,9] Y[ ,10]

1 1 115003 713 923 2101 18616 1230 2278 13687 10420 1481 511

1 2 158559 620 496 1293 14678 3688 1469 13507 10675 1355 400

1 3 123477 674 607 1323 9119 6364 1530 10645 9264 1280 401

2 1 1216654 6606 7403 15412 113099 36185 17311 107417 88600 12442 4810

2 2 1572849 5509 3694 8942 83465 83357 10388 90973 90045 11457 3617

2 3 2111670 9099 7250 15741 101965 196998 18565 142967 139810 18983 5568

3 1 385373 2758 3289 6254 34724 15834 7030 37064 30747 4658 2027

3 2 621887 2651 2541 5357 36275 38837 5942 44238 41011 5378 1761

3 3 1789164 8757 7582 15916 87836 200408 18188 126066 128067 17861 5312

4 1 238594 1824 2398 4218 19261 13569 4676 21948 19075 3104 1358

4 2 252205 1398 1364 2831 16336 19333 3078 21290 18966 2580 919

4 3 1013805 5908 5772 11577 55829 126706 13023 82745 82764 12197 3550

5 1 174923 1405 1707 2993 12723 13479 3302 15801 14083 2421 946

5 2 136807 778 804 1627 8254 13699 1783 11988 11031 1720 498

5 3 408098 2915 2961 5823 24457 59994 6391 38229 38377 5907 1644

6 1 163436 1427 1648 2917 11483 15861 3190 15689 14824 2640 919

6 2 94799 731 611 1235 5942 11969 1327 9174 9043 1486 415

6 3 234193 2287 2125 4127 15906 39234 4490 26012 27011 4540 1147

END
